# Supplementary material for: Mutations as Levy flights
Source: Sci Rep. 2021 May 10;11:9889. doi: 10.1038/s41598-021-88012-1 (PMC8110745; doi:10.1038/s41598-021-88012-1)
Supplement: Supplementary file 1 — Supplementary Information. [file 41598_2021_88012_MOESM1_ESM.pdf]

Supplementary Material for: "Mutations as Levy flights"

Dario A. Leon[1,2,*] **and Augusto Gonzalez**[3,2]

[1]University of Modena & Reggio Emilia, 41125, Modena, Italy
[2]Institute of Cybernetics, Mathematics and Physics, 10400, Havana, Cuba
[3]University of Electronic Science and Technology, 610051, Chengdu, People Republic of China
[*]dario@icimaf.cu

ABSTRACT

We propose a simple method to estimate the mean number of single-nucleotide polymorphisms (SNPs) from the number of fixed point mutations and the frequencies of the non-fixed ones observed in a long-term evolution experiment (LTEE) with E. Coli populations[1].

Estimated mean number of SPMs in a clone

Let us consider a mixed bacterial population, where there are N_{fixed} fixed mutations (frequencies $\geq 96\%$), and a number of additional SNPs with frequencies f_i. We assume that these mutations are not correlated, that is a given frequency f_i is independent from any other f_j. The probability of finding one additional mutation in a clone is, thus:

$$P_1 = \sum_i f_i.$$

If $P(1) > 0.5$ we say that the mean number of mutations we may find in a clone is, at least, $N_{fixed} + 1$.

Similarly, we define:

$$P(2) = \sum_{i<j} f_i f_j,$$

and state that the mean number of mutations is, at least, $N_{fixed} + 2$ if $P(2) > 0.5$.

The probabilities for $N_{fixed} + 3$, $N_{fixed} + 4$, etc mutations are defined in the same way.

We shall say that the mean number of mutations we may find in a clone is $N_{fixed} + n$ if $P(n) > 0.5$, but $P(n+1) < 0.5$.

In order to draw the "model" curve in Fig. 2 of the main manuscript, we use the data of paper[2] for the frequencies of observed SNPs and compute the mean number of mutations in clones.

References

1. Lenski, R. E. Summary data from the Long Term Evolution Experiment. http://myxo.css.msu.edu/ecoli/summdata.html (2019).

2. Barrick, J. E. & Lenski, R. E. Genome-wide mutational diversity in an evolving population of escherichia coli. *Cold Spring Harb. Symp. on Quant. Biol.* **54**, 1, DOI: https://doi.org/10.1101/sqb.2009.74.018 (2009).
